# High Plasma Branched-Chain Amino Acids Are Associated with Higher Risk of Post-Transplant Diabetes Mellitus in Renal Transplant Recipients

**DOI:** 10.3390/jcm9020511

**Published:** 2020-02-13

**Authors:** Maryse C. J. Osté, Jose L. Flores-Guerrero, Eke G. Gruppen, Lyanne M. Kieneker, Margery A. Connelly, James D. Otvos, Robin P. F. Dullaart, Stephan J. L. Bakker

**Affiliations:** 1 Department of Internal Medicine, Division of Nephrology, University Medical Center Groningen, University of Groningen, 9700 RB Groningen, The Netherlands; j.l.flores.guerrero@umcg.nl (J.L.F.-G.); l.m.kieneker@umcg.nl (L.M.K.); s.j.l.bakker@umcg.nl (S.J.L.B.); 2 Department of Internal Medicine, Division of Endocrinology, University Medical Center Groningen, University of Groningen, 9700 RB Groningen, The Netherlands; e.g.gruppen@umcg.nl (E.G.G.); r.p.f.dullaart@umcg.nl (R.P.F.D.); 3 Laboratory Corporation of America Holdings (LabCorp), Morrisville, NC 27560, USA; connem5@labcorp.com (M.A.C.); j.d.otvos@labcorp.com (J.D.O.)

**Keywords:** branched chain amino acids, post-transplant diabetes mellitus, biomarker, renal transplant recipients

## Abstract

Post-transplant diabetes mellitus (PTDM) is a serious complication in renal transplant recipients. Branched-chain amino acids (BCAAs) are involved in the pathogenesis of insulin resistance. We determined the association of plasma BCAAs with PTDM and included adult renal transplant recipients (≥18 y) with a functioning graft for ≥1 year in this cross-sectional cohort study with prospective follow-up. Plasma BCAAs were measured in 518 subjects using nuclear magnetic resonance spectroscopy. We excluded subjects with a history of diabetes, leaving 368 non-diabetic renal transplant recipients eligible for analyses. Cox proportional hazards analyses were used to assess the association of BCAAs with the development of PTDM. Mean age was 51.1 ± 13.6 y (53.6% men) and plasma BCAA was 377.6 ± 82.5 µM. During median follow-up of 5.3 (IQR, 4.2–6.0) y, 38 (9.8%) patients developed PTDM. BCAAs were associated with a higher risk of developing PTDM (HR: 1.43, 95% CI 1.08–1.89) per SD change (*p* = 0.01), independent of age and sex. Adjustment for other potential confounders did not significantly change this association, although adjustment for HbA1c eliminated it. The association was mediated to a considerable extent (53%) by HbA1c. The association was also modified by HbA1c; BCAAs were only associated with renal transplant recipients without prediabetes (HbA1c < 5.7%). In conclusion, high concentrations of plasma BCAAs are associated with developing PTDM in renal transplant recipients. Alterations in BCAAs may represent an early predictive biomarker for PTDM.

## 1. Introduction

Post-transplant diabetes after transplantation (PTDM), often a result of insulin resistance and deficient insulin production [1], is a serious complication in renal transplant recipients [2]. PTDM develops in 10–20% of renal transplant recipients during the first year post-transplantation [3], although some studies reported incidences of up to 50% [4]. PTDM is an important risk factor for cardiovascular disease (CVD) and infections, contributing to impaired graft and patient survival [5,6,7]. Previous studies have shown that PTDM is an important risk factor for premature mortality in renal transplant recipients [8,9,10]. Since the main cause of death in these patients is cardiovascular-related [11,12,13] and most of these patients die with a properly functioning graft [14], it is clinically relevant to identify patients that are at high risk of developing PTDM.

Non-modifiable risk factors for the development of PTDM are patient’s age, race, genetic background, and family history of diabetes. On the other hand, modifiable risk factors include overweight and obesity, but also immunosuppressive medication, such as steroids and calcineurin inhibitors [5]. During the era of cyclosporine-based regimens, the largest number of incident cases of PTDM occurred beyond the first year after transplantation [2] and with current tacrolimus-based regimens, the number of incident cases of PTDM beyond the first year after transplantation is even higher [15]. Furthermore, it should be noted that if a renal transplant recipient is diagnosed with diabetes, it is considered PTDM, irrespective whether this occurs one-year post-transplantation or 10 years later [16].

Current research is focused on a better understanding of risk factors responsible for the development of PTDM. Branched chain amino acids (BCAAs), a group of three essential amino acids (i.e., valine, leucine, and isoleucine), can be obtained from diet and comprise about 15–25% of total protein intake [17]. BCAAs not only play an important role in protein metabolism, but also have metabolic functions [18]. They may stimulate protein synthesis and influence glucose homeostasis [19,20,21]. It is known that circulating concentrations of BCAAs are elevated in subjects with prediabetes, type 2 diabetes, metabolic syndrome, and obesity [20,22,23]. Previous studies have assessed the association of BCAAs with insulin resistance and development of type 2 diabetes [24,25]. Recently, we reported that high concentrations of BCAAs are associated with an increased risk of developing type 2 diabetes in a large prospective cohort study in the general population [26]. High concentrations of BCAAs might be the consequence of excess dietary consumption, dysbiosis of the gut microbiota, and reduced breakdown of BCAAs in skeletal muscle and adipose tissue [20,23,27,28]. It has been found that kidney transplant recipients suffer from dysbiosis of gut microbiota [29,30]. Chronic use of immunosuppressive medication, including glucocorticoids and calcineurin inhibitors, by kidney transplant recipients is moreover likely to influence skeletal muscle metabolism [31,32]. These factors could alter BCAAs in kidney transplant recipients compared to the general population and thereby contribute to development of diabetes.

Previous population-based studies have shown inverse associations between BCAAs and all-cause mortality [33,34,35]. Furthermore, it is known that disturbances in amino acid metabolism, particularly involving BCAAs, occur in patients with end-stage renal disease [36]. A previous study showed that levels of valine and leucine, but not isoleucine, were significantly lower in patients with stage I and II chronic kidney when compared to controls [37].

Whether BCAA plasma concentrations are associated with development of PTDM in renal transplant recipients has not yet been established. Therefore, we hypothesized that higher plasma BCAA concentrations are associated with a higher risk of developing PTDM in renal transplant recipients, as a primary endpoint. Furthermore, secondary endpoints of this study were all-cause mortality and death-censored graft failure, because these endpoints could potentially compete with development of PTDM as an endpoint.

## 2. Materials and Methods

### 2.1. Study Design and Population

In this large cross-sectional study with prospective follow-up, we included stable adult renal transplant recipients (≥18 y) with a functioning graft for at least one year after transplantation (i.e., on maintenance immunosuppression and with a stable renal function), therefore excluding patients with transient hyperglycemia post-transplantation. Between November 2008 and May 2011, patients who visited the outpatient clinic of the University Medical Center Groningen were invited to participate. Both subjects with known or apparent systemic diseases (i.e., malignancies, opportunistic infections) and subjects with a history of alcohol and/or drug addiction were excluded from participation. Informed consent was given by 707 (86.5%) of 817 initially invited patients. We excluded patients with missing data on BCAA, resulting in 518 renal transplant recipients eligible for analyses. As previously described, we recorded age, sex, body composition and eGFR of the renal transplant recipients who did not consent [38]. Compared with participating renal transplant recipients, those who did not consent were slightly older (mean age ± SD, 58 ± 13 years versus 53 ± 13 years) and had lower eGFR (47 ± 19 ml/min per 1.73 m^2^ versus 51 ± 20 ml/min per 1.73 m^2^) [38]. For the analyses with PTDM, we also excluded patients with diabetes or a history of diabetes at baseline (*n* = 132). Of these 132 renal transplant recipients, 34 were diagnosed with diabetes before renal transplantation and 98 developed PTDM between time of transplantation and baseline, leaving 386 renal transplant recipients eligible for analyses (Supplementary Figure S1). The study protocol was approved by the institutional research board (METc 2008/186), which adheres to the Principles of the Declaration of Helsinki.

### 2.2. Data Collection

During a morning visit to the outpatient clinic, all baseline data were collected as described previously [39]. Body weight and height were measured. Body Mass Index (BMI) was calculated as weight in kilograms divided by height in meters squared (kg/m^2^). Systolic and diastolic blood pressure and heart rate were measured every minute for 15 minutes in a half-sitting position using a semi-automatic device (Dinamap^®^1846; Critikon, Tampa, FL, USA) to prevent white coat effects [40]. The average of the last three measurements was taken as blood pressure value. Information on medication was derived from patient records, whereas information on smoking behavior was obtained by questionnaire. Information on physical activity was obtained using the reliable and valid Short Questionnaire to Assess Health enhancing physical activity (SQUASH) score in time multiplied by intensity [41]. Alcohol consumption and total energy intake were measured using a reproducible, validated food frequency questionnaire (FFQ) [42], which consisted of 177 items and was updated several times. Blood samples were taken after an 8–12 h overnight fasting period in the morning after completion of 24 h urine collection. Renal transplant recipients were instructed to assure adequate urine collection. They were instructed to discard their first morning urine specimen and then collect their urine for the next 24 h, including the next morning’s first specimen the day of their visit. Protein intake was measured using 24 h urinary urea excretion. Estimated Glomerular Filtration Rate (eGFR) was calculated using the serum creatinine-based Chronic Kidney Disease Epidemiology Collaboration equation [43].

### 2.3. Quantification of BCAAs

Plasma BCAA concentrations were measured at baseline using a Vantera Clinical Analyzer (LabCorp, Morrisville, NC, USA), a fully automated, high-throughput, 400 MHz proton (^1^H) nuclear magnetic resonance spectroscopy (NMR) platform. Quantification of BCAAs by NMR was validated and had been previously described in detail elsewhere [25,44]. The within-laboratory (inter-assay) and within-run (intra-assay) imprecision for NMR-measured BCAAs are described in detail previously [44]. For total BCAAs, the coefficients of variation for inter-assay and intra-assay were 1.8–6.0% and 2.1–4.4%, respectively.

### 2.4. Clinical Endpoints

The primary outcome of this study was PTDM, which was defined as at least one of the following criteria: symptoms of diabetes (e.g., polyuria, polydipsia, unexplained weight loss) plus a non-fasting plasma glucose concentration of ≥200mg/dL (11.1 mmol/L); fasting plasma glucose concentration (FPG) ≥126 mg/dL (7.0 mmol/L); start of antidiabetic medication; or HbA1c ≥6.5% (48 mmol/L). This definition was according to the American Diabetes Association criteria for diabetes [45], including HbA1c levels as proposed by the International Expert Panel of the international consensus meeting on PTDM [46]. The secondary outcomes of this study were all-cause mortality and death-censored graft failure. Death-censored graft failure was defined as return to hemodialysis treatment or retransplantation. All subjects received medical care at the University Medical Center Groningen alone or medical care shared with a secondary referral hospital. In accordance with the KDIGO guideline for renal transplant recipients, follow-up visits after the first year post transplantation were performed every 3 months [47]. Data on PTDM, all-cause mortality and death-censored graft failure were retrieved from patient files and verified with the corresponding nephrologist or the Municipal Personal Records Database in case of death. Endpoints were recorded until the end of September 2015. Since the outpatient program uses continuous surveillance systems, it guarantees correct and up-to-date information on patient status. No participants were lost to follow-up.

### 2.5. Statisical Analyses

Normal distributed data were presented as mean and standard deviation, whereas skewed distributed data were expressed as median and interquartile range. Categorical data were presented as number and percentage. Differences between diabetic and non-diabetic renal transplant recipients were compared using unpaired t-tests for normally distributed variables, Mann–Whitney U tests for skewed distributed variables and Chi-square tests for categorical variables. Differences between tertiles of total BCAA were compared using one-way ANOVA tests for normally distributed variables, Kruskal–Wallis tests for skewed distributed variables and Chi-square tests for categorical variables. Skewed distributed data were log-transformed when appropriate. Correlations between BCAAs and total energy intake, protein intake, physical activity, and HbA1c in non-diabetic renal transplant recipients were assessed using Pearson correlation coefficients.

Kaplan–Meier curves were plotted for the development of PTDM according to the highest tertile versus the two lowest tertiles of total BCAA. We performed crude and multivariable Cox proportional hazards regression analyses to assess the association of total BCAA with the development of PTDM. First, we performed crude analyses and analyses adjusted for age and sex (model 1). We further cumulatively adjusted for renal function parameters (eGFR, proteinuria, and time between transplantation and baseline) in model 2. To prevent overfitting by including too many covariates in relation to number of events [48], we adjusted for other potential confounders in additional models based on model 2. We additionally adjusted for total cholesterol and triglycerides in model 3; total energy intake, physical activity, and BMI in model 4; smoking status and alcohol consumption in model 5; prednisolone dose and trough levels of tacrolimus and cyclosporine in model 6. Total BCAA per 1 standard deviation (SD) was used as continuous variable, but also as categorical variable (highest tertile versus two lowest tertiles). Patients were censored at date of last follow-up or death. Hazards ratios and 95% CIs were given for the Cox proportional hazards analyses. Schoenfeld residuals of the BCAAs were checked and tested in STATA using the proportional hazard test by Grambsch and Therneau [49]. Furthermore, penalized splines analyses performed in R were used to visualize the association of total BCAA with the development of PTDM, adjusted for age and sex. Additionally, we evaluated potential effect modification by age, gender, BMI, eGFR and HbA1c by entering both main effects and the cross-product term in the crude model. When effect modification was observed, we proceeded with stratified analyses, with a HbA1c of 5.7–6.4% considered as prediabetic state [50,51].

In further analyses, we investigated whether plasma glucose and HbA1c could serve as mediator in the association of BCAAs and risk of PTDM. To investigate potential mediation, we performed mediation analyses using the mediation package of R [52], by which we tested significance and magnitude of mediation (see the Supplementary Materials for a detailed description). Competing risks occur when patients can experience or develop one or more events which compete with the outcome of interest [53]. To rule out competing risk of all-cause mortality with the development of PTDM, we performed competing risk analyses according to Fine and Gray [54]. We performed crude and multivariable Cox proportional hazards regression analyses to assess the association of total BCAA with the secondary outcomes all-cause mortality and death-censored graft failure. Total BCAA per 1 standard deviation (SD) was used as continuous variable, but also as categorical variable (lowest tertile versus two highest tertiles). For the association of BCAAs with both all-cause mortality and death-censored graft failure, we evaluated the potential effect modification by diabetes.

A two-sided *p*-value of <0.05 was considered statistically significant. The main statistical analyses for the manuscript were performed using IBM Statistics SPSS version 23.0 (IBM Inc, Chicago, IL, USA). We used STATA version 11.0 (StataCorp LP, College Station, TX, USA) to check and test the Schoenfeld residuals by performing the proportional hazard test according to Grambsch and Therneau. We used R version 3.2.3 (R Foundation for Statistical Computing, Vienna, Austria) to perform penalized splines analyses and to perform mediation analyses. We used GraphPad Prism 5 (GraphPad Software Inc., La Jolla, CA, USA) to visualize the Kaplan–Meier curves for the development of PTDM.

## 3. Results

### 3.1. Patient Characteristics in Whole Cohort (n = 518)

Mean age of overall renal transplant recipients was 52.7 ± 13.0 y and 53.7% of the participants were men. Median time between baseline measurements and transplantation was 5.0 (IQR, 1.7–11.9) years. Diabetic renal transplant recipients had significantly higher plasma concentrations of total BCAA (424.6 ± 97.9 µM) when compared with non-diabetic renal transplant recipients (377.6 ± 82.5 µM). Baseline characteristics of the overall (*n* = 518), diabetic (*n* = 132) and non-diabetic (*n* = 386) population are shown in Table 1. Non-diabetic subjects were younger, had a lower weight and BMI, had a higher physical activity score and lower heart rate when compared with diabetic RTR. Furthermore, non-diabetic subjects had lower plasma glucose, HbA1c, and triglycerides, and higher HDL cholesterol concentrations. No differences were seen in medication, except for use of statins, which was more common in the diabetic renal transplant recipients than in the non-diabetic renal transplant recipients.

### 3.2. Patient Characteristics in Subgroup of Non-Diabetic Renal Transplant Recipients (n = 386)

For the non-diabetic renal transplant recipients the baseline characteristics according to tertiles of total BCAAs are presented in Table 2. Subjects in the highest tertile of total BCAA were more often male, consumed more alcohol, had a lower heart rate, and a lower HDL cholesterol when compared with subjects in the lowest tertile. There were no differences in transplant characteristics, renal allograft function, and glucose homeostasis. Furthermore, we found that total BCAAs were positively correlated with protein intake (*r* = 0.25, *p* = <0.001) and HbA1c (*r* = 0.12, *p* = 0.02), but not with total energy intake (*r* = –0.01, *p* = 0.82) and physical activity (*r* = 0.10, *p* = 0.06). When we divided the non-diabetic renal transplant recipients in patients with prediabetes (HbA1c ≥ 5.7%) and without prediabetes (HbA1c < 5.7%), we found a positive correlation in the prediabetic renal transplant recipients (*r* = 0.23, *p* = 0.002), but not in renal transplant recipients without prediabetes (*r* = 0.003, *p* = 0.96).

### 3.3. BCAAs and Risk of Developing PTDM

**Figure 1:**
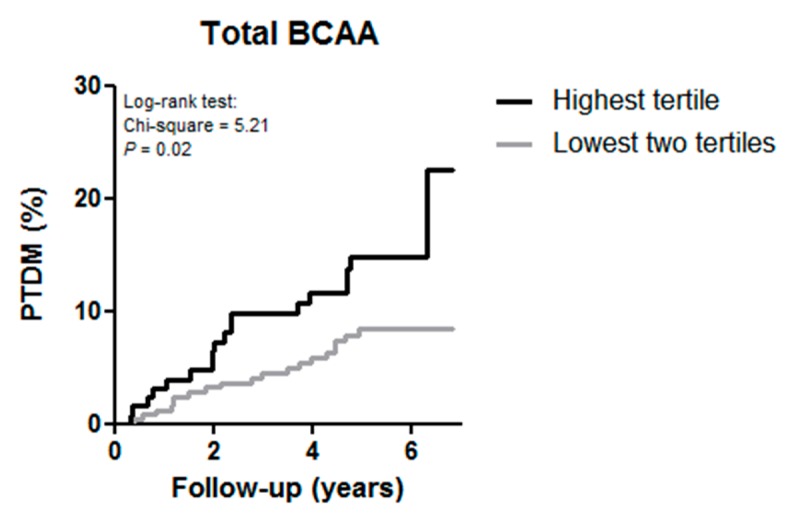
Kaplan–Meier curves for the development of post-transplant diabetes mellitus (PTDM) according to the highest tertile versus the two lowest tertile of total branched chain amino acids (BCAA) in renal transplant recipients.

In the subgroup of non-diabetic renal transplant recipients at baseline (*n* = 386) during a median follow-up of 5.3 (IQR, 4.2–6.0) y, 38 (9.8%) subjects developed PTDM. Of the renal transplant recipients in the highest tertile of total BCAA 17.3% developed PTDM versus 8.0% in the lowest two tertiles (*p* = 0.02). The Kaplan–Meier curves for the development of PTDM according to the highest tertile versus the two lowest tertiles of total BCAA is shown in Figure 1.

**Figure 2:**
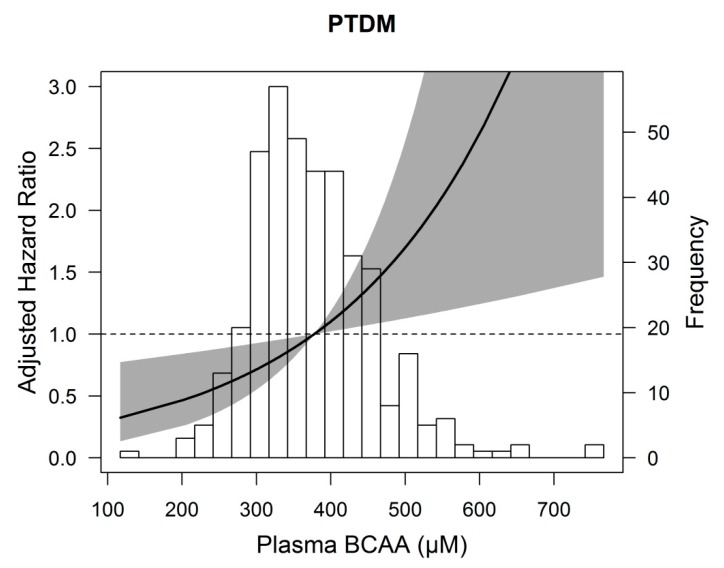
Association between plasma branched chain amino acids (BCAA) and post-transplant diabetes mellitus (PTDM) in 386 renal transplant recipients. Data were fit by a Cox regression model based on penalized splines and adjusted for age and sex. The gray area represents the 95% confidence interval.

Cox regression analyses with total BCAA per standard deviation (SD) as continuous variable showed that higher total BCAA was associated with a higher risk of developing PTDM (HR: 1.43, 95% CI 1.08–1.89, *p* = 0.01), independent of age and sex (Table 3, model 1). After adjustment for other potential confounders, including renal function parameters, lipids, dietary and lifestyle factors, and use of medication the association did not materially change (Table 3, model 2–6). In additional Cox regression analyses with total BCAA divided in the highest tertile versus the two lower tertiles, total BCAA was again significantly associated with development of PTDM, independent of age and sex (HR: 2.07; 95% CI 1.07–3.99, *p* = 0.03). Further adjustment for potential confounders did not change the association (Table 3, model 2–6). To illustrate the association of total BCAA with development of PTDM, an age and sex adjusted penalized spline is shown in Figure 2. We found no significant effect modification by age (*p*_interaction_ = 0.75), gender (*p*_interaction_ = 0.17), BMI (*p*_interaction_ = 0.31), and eGFR (*p*_interaction_ = 0.50) in the association of total BCAA per SD with PTDM, but we did for HbA1c (*p*_interaction_ = 0.02). We continued with stratified analyses (Supplementary Figure S2). BCAAs were associated with PTDM in renal transplant recipients without prediabetes (HbA1c < 5.7%), but not in renal transplant recipients with prediabetes (HbA1c ≥ 5.7%), independent of age, sex, eGFR, proteinuria, and time since transplantation.

### 3.4. Secondary Analyses

In mediation analyses, we found that HbA1c mediated 53% of the association between BCAAs and PTDM in renal transplant recipients, whereas plasma glucose was not a significant mediator in this association (Supplementary Table S1), after adjustment for age and sex. The results of competing risk analyses did not materially differ from those with Cox regression for the association of total BCAA per SD as continuous variable and development of PTDM (HR: 1.44, 95% CI 1.08–1.92, *p* = 0.01), adjusted for age and sex (Table 3, model 2 for comparison). Also, the analysis with total BCAA divided in the highest tertile versus the two lower tertiles did not differ in the competing risk analysis (HR: 2.09, 95% CI 1.10–3.96, *p* = 0.02), adjusted for age and sex (Table 3, model 2 for comparison).

### 3.5. BCAAs and Risk of All-Cause Mortality and Death-Censored Graft Failure

In the total population of both diabetic and non-diabeticrenal transplant recipients at baseline (*n* = 518), 114 (22.0%) subjects died during a median follow-up of 5.4 (IQR, 4.7–6.2) y, whereas 65 (12.5%) subjects developed graft failure during a median follow-up of 5.3 (IQR, 4.5–6.0) y. There was no significant association between total BCAA and the individual BCAAs with all-cause mortality and death-censored graft failure (Supplementary Table S2). We found no effect modification by diabetes for the association of total BCAA with all-cause mortality (*p*_interaction_ = 0.22) and death-censored graft failure (*p*_interaction_ = 0.41). 

## 4. Discussion

In this large cross-sectional study with prospective follow-up, higher concentrations of total BCAAs are associated with a higher risk of developing PTDM in renal transplant recipients. This association did not change after adjustment for relevant confounders, including age, sex, renal function parameters, lipids, dietary and lifestyle factors, and use of immunosuppressive medication. Subsequently, this association was modified by HbA1c; total BCAAs were significantly associated with PTDM in renal transplant recipients without prediabetes (HbA1c < 5.7%), but not in renal transplant recipients with prediabetes (HbA1c ≥ 5.7%). Furthermore, we show that the association between total BCAA and PTDM was mediated to a considerable extent (53%) by HbA1c. In addition, no association of total BCAAs with all-cause mortality and death-censored graft failure in renal transplant recipients was found.

It is known that BCAAs are elevated in subjects with prediabetes, type 2 diabetes, and obesity [22]. In this cohort, BCAA concentrations were elevated in diabetic renal transplant recipients when compared with non-diabetic renal transplant recipients (424.6 ± 97.9 µM vs. 377.6 ± 82.5 µM, respectively), as observed in previous studies in the general population [23,55,56]. The BCAA concentrations of the diabetic renal transplant recipients can be compared to the BCAA concentrations of 439 ± 95 µM in patients with type 2 diabetes mellitus in the general population [25]. The BCAA concentrations of the non-diabetic renal transplant recipients are comparable to the mean plasma levels of 370.3 ± 88.6 µM in a large prospective population-based cohort study [26].

BCAAs are a group of essential amino acids, comprising valine, leucine, and isoleucine, and can only be obtained from diet. They comprise about 15–25% of total protein intake [17]. Previous studies have shown that plasma BCAA levels are modifiable by a higher or lower consumption of protein. Prior work showed that higher consumption of BCAAs is significantly associated with higher plasma levels of BCAAs [57]. The correlation was moderate, but comparable to other diet-plasma biomarker correlations. It has been shown that dietary protein reduction lowers serum levels of BCAAs [58]. Recently, a randomized controlled crossover trial even showed that short term dietary reduction of BCAAs decreases postprandial insulin secretion [59]. It is known, that around 80% of dietary BCAAs reach the blood circulation [60], but circulating plasma levels of BCAAs can also be affected by their catabolism [61]. The initial site of the BCAA metabolism is skeletal muscle, because of the high branched-chain-amino-acid aminotransferase (BCAT) activity in the muscle [62]. This metabolism is sensitive to changes in the amount and composition of food. A high protein diet leads to higher concentrations of BCAAs, whereas a low protein diet lowers the plasma BCAA concentrations [62,63]. Indeed, in our study, subjects in the highest tertile of total BCAA had a higher 24 h urinary urea excretion, which is an objective measurement for total protein intake, when compared to subjects in the lowest tertile of total BCAA.

The results of the prospective analysis with PTDM are consistent with previous studies that reported the association of BCAAs with type 2 diabetes in the general population [21,22,64]. Recently, we showed in a prospective cohort study that high concentrations of BCAAs are associated with increased risk of developing type 2 diabetes [26]. The fact that total BCAAs were significantly associated with PTDM in subjects without prediabetes, but not in prediabetic subjects suggest that alterations in total BCAAs might be an early signal of deterioration of glycemic control. A previous study has shown that elevated BCAAs levels may appear long before other markers of insulin resistance become abnormal [21]. Elevations in circulating BCAAs can occur before any alterations in insulin action are detectable. Moreover, it has been reported that plasma BCAAs might serve as a better indicator of impaired insulin resistance when compared to plasma glucose levels [65], since in patients without prediabetes the metabolic status is not deteriorated enough to alter plasma glucose levels.

The secondary outcomes, all-cause mortality and death-censored graft failure, were not associated with total BCAAs. This is in contrast to a previous study that showed an inverse association of total BCAAs and death in patients at risk for coronary artery disease [33], supporting the underlying malnutrition-inflammation syndrome hypothesis. Furthermore, the large Estonian biobank study also observed inverse associations between BCAAs and all-cause mortality [34]. Moreover, in the ADVANCE study including individuals with type 2 diabetes, low levels of leucine and valine were associated with increased all-cause mortality [35]. In our study 114/518 (22.0%) renal transplant recipients died during a median follow-up of 5.4 years, resulting in a death rate of 4.1% per year, which is slightly higher when compared to the FAVORIT trial, a large multi-center double-blind randomized controlled trial in 4110 stable renal transplant recipients (age 52 ± 9.4 years, 62.8% male at 5 years after transplantation) in which 493/4110 (12.0%) subjects died within 4.0 years of follow-up, resulting in a death rate of 3.0% per year [66]. Prior work showed that levels of valine and leucine, but not isoleucine, were significantly lower in patients with stage I and II chronic kidney disease, when compared with controls [37], suggesting potential use as a biomarker for renal dysfunction.

Currently, there are several potential mechanisms that could explain the contribution of BCAAs to the development of insulin resistance, type 2 diabetes, and PTDM, although these mechanisms are not completely understood. One mechanism proposes that BCAAs interfere with insulin signaling through activation of the mammalian target of rapamycin complex 1 (mTORC1) in skeletal muscle and serine phosphorylation of insulin receptor substrate 1 and 2, which promotes insulin resistance and can lead to the development of type 2 diabetes [20]. However, conflicting results regarding the role of BCAAs to elicit insulin resistance have been reported [67] and do question whether mTORC1 activation is sufficient or necessary in the development of insulin resistance. Others assume that BCAA dysmetabolism, especially in obesity, contributes to a rise in BCAAs, which results in accumulation of potential toxic BCAA metabolites, which could induce cellular damage [20]. These BCAA metabolites might lead to mitochondrial dysfunction and β-cell apoptosis, which is common in insulin resistance and type 2 diabetes [20]. Nevertheless, the association of total BCAAs and PTDM in our study was independent of BMI. Moreover, a previous study in the general population showed that the association of BCAAs with insulin resistance was independent of leptin and adiponectin, both valid biomarkers of adipose tissue dysfunction, when taking BMI into account [25], suggesting the association is presumably mainly driven by another mechanism.

To the best of our knowledge, this is the first study that studied the association of BCAAs with the development of PTDM in renal transplant recipients. Strengths of this study include the complete follow-up and use of clinical endpoints (PTDM, all-cause mortality, and death-censored graft failure), which are relevant in daily clinical practice. Furthermore, this study had a considerable follow-up period. Another strength is the use of stable patients who had a functioning graft for at least 1 year, which resulted in exclusion of patients with transient hyperglycemia post-transplantation, which occurs frequently and is evident in about 90% of renal transplant recipients in the first few weeks post-transplantation [68,69]. Hyperglycemia can also occur as a result of rejection therapy, infections, and other critical conditions [46]. Therefore, it is important to diagnose PTDM in stable patients (i.e., on maintenance immunosuppression, stable renal function and in absence of acute infections) [46]. This study also has several limitations. First, it is a single-center study, with a study population mainly consisting of Caucasians. As ethnicity is an independent risk factor for developing PTDM [5], it is important to repeat this study in more diverse populations. Renal transplant recipients in the current study were included at a median of 5.0 years after transplantation. Therefore, extrapolating our results to patients in early stages after renal transplantation should be done with caution. The clinical significance or impact of the diagnosis of PTDM early or late after transplantation has yet to be determined [16]. In addition, age at time of transplantation in our cohort is lower when compared to other European cohorts [70]. Moreover, the prevalence of living donor grafts is higher in the Netherlands [71], which might also contribute to a lower age in our cohort, since younger subjects have a broader social network and therefore likely a higher chance of finding a compatible living donor at younger age. Furthermore, it is known that oral glucose tolerance tests (OGTTs) are the gold standard diagnostic tool to diagnose PTDM. Unfortunately, OGTTs were not performed, but recently it has been shown that the combined use of fasting plasma glucose and HbA1c criteria appears to be a diagnostic strategy for PTDM in stable renal transplant recipients [72]. Another limitation of this study is that only 38 subjects developed PTDM during follow-up, which led to a lack of power. Unfortunately, a comorbidity index was not available in our cohort and we do not have data on weight gain post-transplantation. It cannot be excluded that change of weight could serve as a source of bias and could spuriously strengthen or weaken the association of BCAAs with development of PTDM. Finally, longer-term intervention studies are required to determine whether BCAAs are causally related to the development of diabetes mellitus or merely act as markers of underlying pathophysiology.

## 5. Conclusions

In conclusion, this single-center cross-sectional assessment of BCAA in stable renal transplant recipients showed that high plasma concentrations of total BCAA are associated with a higher risk of developing PTDM during prospective follow-up. Alterations in BCAA levels might be an early signal of deterioration of glycemic control in renal transplant recipients. Further research is needed to investigate the possible mechanism/role of BCAAs in the development of post-transplant diabetes.

## Figures and Tables

**Table 1:** Baseline characteristics in renal transplant recipients (*n* = 518).

	Total (*n* = 518)	Diabetic RTR (*n* = 132)	Non-Diabetic RTR (*n* = 386)	*P*-Value
**General characteristics**				
Age, years	52.7 ± 13.0	57.2 ± 10.1	51.1 ± 13.6	<0.001
Male sex, *n* (%)	278 (53.7)	71 (46.2)	207 (53.6)	0.97
Race (white), *n* (%)	515 (99.4)	132 (100.0)	383 (99.2)	0.31
BMI, kg/m^2^	26.6 ± 4.8	28.7 ± 5.3	25.9 ± 4.5	<0.001
Physical activity score (time x intensity)	5075 (2190–8100)	3690 (1215–6563)	5590 (2810–8715)	<0.001
Smoking status, *n* (%)				0.16
Never	203 (39.2)	49 (37.1)	154 (39.9)	
Former	228 (44.0)	63 (47.7)	165 (42.7)	
Current	63 (12.2)	10 (7.6)	53 (13.7)	
Alcohol consumption, g/d	2.3 (0.0–11.1)	1.6 (0.0–7.4)	2.6 (0.0–11.7)	0.05
Total energy intake, kcal/d	2139 ± 634	2106 ± 599	2152 ± 647	0.49
Urea excretion, mmol/24h	389.4 ± 117.2	391.2 ± 124.3	388.8 ± 114.9	0.83
**Circulation**				
Heart rate, b.p.m.	68.6 ± 12.1	72.2 ± 11.3	67.3 ± 12.1	<0.001
SBP, mmHg	135.9 ± 17.7	138.2 ± 18.5	135.1 ± 17.4	0.07
DBP, mmHg	82.1 ± 11.0	82.0 ± 10.3	82.1 ± 11.3	0.93
**Transplant characteristics**				
Transplant vintage, years	5.0 (1.7–11.9)	6.2 (1.7–11.8)	4.9 (1.7–12.0)	0.59
Living donor, *n* (%)	182 (35.1)	37 (28.0)	145 (37.6)	0.05
Pre-emptive transplant, *n* (%)	91 (17.6)	14 (10.6)	77 (19.9)	0.02
Dialysis duration, months	42.0 (18.5–59.0)	36.0 (18.5–52.5)	44.0 (17.0–59.8)	0.58
Age donor, years	43.7 ± 15.2	41.9 ± 15.2	44.3 ± 15.2	0.12
**Renal allograft function**				
Serum creatinine, µmol/L	127.0 (101.0–167.3)	133.0 (102.3–166.0)	126.0 (101.0–168.0)	0.77
eGFR, ml/min per 1.73 m^2^	50.6 ± 19.9	49.5 ± 20.8	51.0 ± 19.6	0.45
Proteinuria, *n* (%)	117 (22.6)	35 (26.5)	82 (21.2)	0.19
**Glucose homeostasis**				
Plasma glucose (mmol/L)	5.2 (4.8–6.0)	7.0 (5.4–8.1)	5.1 (4.7–5.5)	<0.001
HbA1c (%)	6.0 ± 0.8	6.9 ± 1.1	5.7 ± 0.4	<0.001
**Lipids and lipoproteins**				
Total cholesterol, mmol/L	5.2 ± 1.2	5.2 ± 1.2	5.1 ± 1.1	0.64
HDL-cholesterol, mmol/L	1.4 ± 0.5	1.3 ± 0.4	1.4 ± 0.5	0.03
LDL-cholesterol, mmol/L	3.0 ± 0.9	3.0 ± 1.0	3.0 ± 0.9	0.59
Triglycerides, mmol/L	1.7 (1.3–2.4)	1.9 (1.4–3.0)	1.7 (1.2–2.2)	<0.001
**Medication**				
Calcineurin inhibitor, *n* (%)				0.19
Cyclosporine	218 (42.1)	61 (46.2)	157 (40.7)	
Tacrolimus	93 (18.0)	27 (20.5)	66 (17.1)	
Trough level cyclosporine (µg/L)	108.0 (77.0–144.0)	102.5 (74.0–156.0)	111.0 (77.5–142.0)	0.78
Trough level tacrolimus (µg/L)	6.8 (5.0–9.0)	6.6 (5.4–9.9)	7.2 (4.9–9.0)	0.78
Proliferation inhibitor, *n* (%)				0.20
Azathioprine	100 (19.3)	22 (16.7)	78 (20.2)	
Mycofenol	333 (64.3)	82 (62.1)	251 (65.0)	
Prednisolone, *n* (%)	513 (99.0)	131 (99.2)	382 (99.0)	0.78
Prednisolone dose, mg/24h	10.0 (7.5–10.0)	10.0 (7.5–10.0)	10.0 (7.5–10.0)	0.71
Antihypertensive drugs, *n* (%)	462 (89.2)	121 (91.7)	341 (88.3)	0.29
Statins, n (%)	270 (52.1)	84 (63.6)	186 (48.2)	0.002
**Amino acids**				
Total BCAA, µM	389.6 ± 89.0	424.6 ± 97.9	377.6 ± 82.5	<0.001
Valine, µM	203.0 ± 44.7	217.0 ± 48.9	198.2 ± 42.2	<0.001
Leucine, µM	141.7 ± 37.3	157.1 ± 42.1	136.5 ± 34.0	<0.001
Isoleucine, µM	44.9 ± 19.1	51.8 ± 20.0	43.5 ± 17.8	<0.001

Data are represented as mean ± SD, median (interquartile range) or *n* (%). Differences were tested by unpaired T-test or Mann–Whitney U test for continuous variables and with χ ^2^-test for categorical variables. RTR, renal transplant recipients; SBP, systolic blood pressure; DBP, diastolic blood pressure; eGFR, estimated glomerular filtration rate; HbA1c, hemoglobin A1c; LP-IR, lipoprotein insulin resistance index; HDL, high-density lipoprotein; LDL, low-density lipoprotein; BCAA, branched-chain amino acids.

**Table 2:** Baseline characteristics of non-diabetic renal transplant recipients according to tertiles of total BCAA (*n* = 386).

	Tertile 1 (*n* = 127)	Tertile 2 (*n* = 130)	Tertile 3 (*n* = 129)	*P*-Value
**General characteristics**				
Age, years	49.9 ± 13.3	52.8 ± 14.4	50.6 ± 12.9	0.19
Male sex, *n* (%)	81 (63.8)	73 (56.2)	88 (68.2)	<0.001
Race (white), *n* (%)	126 (99.2)	129 (99.2)	128 (99.2)	1.00
BMI, kg/m^2^	25.4 ± 5.2	26.0 ± 4.2	26.3 ± 3.9	0.27
Physical activity score (time x intensity)	4930 (2100–7260)	5905 (3315–8625)	6100 (3120–9860)	0.05
Smoking status, *n* (%)				0.10
Never	54 (42.5)	58 (44.6)	42 (32.6)	
Former	47 (37.0)	51 (39.2)	67 (51.9)	
Current	21 (16.5)	17 (13.1)	15 (11.6)	
Alcohol consumption, g/d	1.6 (0.0–8.9)	2.9 (0.1–11.3)	4.3 (0.1–15.8)	0.03
Total energy intake, kcal/d	2178 ± 631	2184 ± 724	2096 ± 578	0.51
Urea excretion, mmol/24h	335.1 ± 92.5	406.6 ± 117.2	423.2 ± 114.2	<0.001
**Circulation**				
Heart rate, b.p.m.	70.3 ± 12.3	66.0 ± 12.5	65.8 ± 11.0	0.005
SBP, mmHg	134.9 ± 17.8	133.9 ± 18.2	136.4 ± 16.1	0.51
DBP, mmHg	81.7 ± 11.8	80.4 ± 11.4	84.1 ± 10.4	0.03
**Transplant characteristics**				
Transplant vintage, years	5.0 (1.8–14.9)	4.7 (1.7–12.0)	4.9 (1.3–10.8)	0.37
Living donor, *n* (%)	56 (44.1)	39 (30.0)	50 (38.8)	0.06
Pre-emptive transplant, *n* (%)	33 (26.0)	22 (16.9)	22 (17.1)	0.12
Dialysis duration, months	34.5 (11.0–63.0)	47.0 (14.0–60.5)	37.0 (22.5–58.5)	0.81
Age donor, years	42.7 ± 15.5	44.7 ± 15.6	45.5 ± 14.4	0.33
**Renal allograft function**				
Serum creatinine, µmol/L	124.0 (98.0–175.0)	123.0 (99.8–154.5)	134.0 (104.0–180.5)	0.23
eGFR, ml/min per 1.73 m^2^	50.1 ± 21.1	52.8 ± 19.3	50.1 ± 18.2	0.44
Proteinuria, *n* (%)	28 (22.0)	28 (21.5)	26 (20.2)	0.92
**Glucose homeostasis**				
Plasma glucose (mmol/L)	5.1 (4.6–5.5)	5.1 (4.7–5.4)	5.0 (4.7–5.6)	1.00
HbA1c (%)	5.6 ± 0.3	5.6 ± 0.4	5.7 ± 0.4	0.10
**Lipids and lipoproteins**				
Total cholesterol, mmol/L	5.2 ± 1.0	5.1 ± 1.1	5.2 ± 1.3	0.62
HDL-cholesterol, mmol/L	1.5 ± 0.5	1.4 ± 0.4	1.3 ± 0.4	<0.001
LDL-cholesterol, mmol/L	3.0 ± 0.8	3.0 ± 1.0	3.1 ± 1.0	0.43
Triglycerides, mmol/L	1.6 (1.2–2.1)	1.6 (1.2–2.2)	1.7 (1.3–2.4)	0.19
**Medication**				
Calcineurin inhibitor, *n* (%)				0.57
Cyclosporine	53 (41.7)	54 (41.5)	50 (38.8)	
Tacrolimus	19 (15.0)	19 (14.6)	28 (21.7)	
Trough level cyclosporine (µg/L)	112.0 (78.3–143.3)	102.0 (74.8–141.5)	105.0 (74.5–156.5)	0.85
Trough level tacrolimus (µg/L)	5.5 (3.9–8.0)	7.7 (6.0–9.7)	7.4 (6.0–9.6)	0.08
Proliferation inhibitor, *n* (%)				0.04
Azathioprine	34 (26.8)	25 (19.2)	19 (14.7)	
Mycofenol	71 (55.9)	84 (64.6)	96 (74.4)	
Prednisolone, *n* (%)	127 (100.0)	128 (98.5)	127 (98.4)	0.37
Prednisolone dose, mg/24h	10.0 (7.5–10.0)	10.0 (7.5–10.0)	10.0 (7.5–10.0)	0.19
Antihypertensive drugs, *n* (%)	107 (84.3)	117 (90.0)	117 (90.7)	0.21
Statins, *n* (%)	53 (41.7)	66 (50.8)	67 (51.9)	0.20
**Amino acids**				
Total BCAA, µM	297.1 ± 33.7	366.6 ± 20.4	467.9 ± 64.7	<0.001
Valine, µM	159.1 ± 20.7	194.5 ± 18.4	240.5 ± 35.3	<0.001
Leucine, µM	107.6 ± 21.5	133.3 ± 17.3	168.2 ± 29.3	<0.001
Isoleucine, µM	31.6 ± 10.0	39.1 ± 11.2	59.1 ± 18.0	<0.001

Data are represented as mean ± SD, median (interquartile range) or *n* (%). Differences were tested by analysis of variance or Kruskal–Wallis for continuous variables and with χ ^2^-test for categorical variables. SBP, systolic blood pressure; DBP, diastolic blood pressure; eGFR, estimated glomerular filtration rate; HbA1c, hemoglobin A1c; LP-IR, lipoprotein insulin resistance index; HDL, high-density lipoprotein; LDL, low-density lipoprotein; BCAA, branched-chain amino acids.

**Table 3:** Association of plasma BCAAs with post-transplant diabetes mellitus in renal transplant recipients (*n* = 386).

	Per SD as Continuous Variable (µmol/L)		Highest Tertile vs. Lower Two Tertiles		
BCAA					
No. of events	38		19	19	
	HR (95% CI)	*P*	Reference	HR (95% CI)	*P*
Crude	1.43 (1.09–1.88)	0.009	1.00	2.06 (1.09–3.90)	0.03
Model 1	1.43 (1.08–1.89)	0.01	1.00	2.07 (1.07–3.99)	0.03
Model 2	1.43 (1.07–1.90)	0.02	1.00	1.97 (1.02–3.82)	0.05
Model 3	1.37 (1.03–1.84)	0.03	1.00	1.82 (0.93–3.57)	0.08
Model 4	1.42 (1.06–1.90)	0.02	1.00	1.90 (0.95–3.80)	0.07
Model 5	1.47 (1.10–1.96)	0.009	1.00	2.09 (1.05–4.17)	0.04
Model 6	1.42 (1.08–1.85)	0.01	1.00	2.12 (1.09–4.12)	0.03

Cox proportional hazards regression analyses were performed to assess the association of BCAAs with PTDM. Model 1: adjustment for age and sex; model 2: model 1 + adjustment for eGFR, proteinuria, and time since transplantation; model 3: model 2 + adjustment for total cholesterol and triglycerides; model 4: model 2 + adjustment for total energy intake, physical activity, and BMI; model 5: model 2 + adjustment for smoking status and alcohol intake; model 6: model 2 + adjustment for prednisolone dose and trough levels of tacrolimus and cyclosporine;. BCAA, branched chain amino acids; PTDM, post-transplant diabetes mellitus; eGFR, estimated glomerular filtration rate.

## Supplementary Materials

The following are available online at https://www.mdpi.com/2077-0383/9/2/511/s1, Figure S1: Flowchart of the study, Figure S2: Stratified analyses of the association of branched chain amino acids (BCAA) and post-transplant diabetes mellitus (PTDM) in both patients with normal glucose tolerance (HbA1c < 5.7%) and prediabetes (HbA1c ≥ 5.7%) adjusted for age, sex, eGFR, proteinuria, and time since transplantation, Figure S3: Mediation analysis on the association of branched chain amino acids (BCAA) on post-transplant diabetes mellitus (PTDM), Table S1: Mediating effect of HbA1c on the association of BCAA with PTDM, Table S2: Association of BCAAs with all-cause mortality and death-censored graft failure in renal transplant recipients (*n* = 518).
