# Supplementary material for: High Plasma Branched-Chain Amino Acids Are Associated with Higher Risk of Post-Transplant Diabetes Mellitus in Renal Transplant Recipients
Source: J Clin Med. 2020 Feb 13;9(2):511. doi: 10.3390/jcm9020511 (PMC7073569; doi:10.3390/jcm9020511)
Supplement: Supplementary file 1 [file jcm-09-00511-s001.pdf]

Supplementary Material

High Plasma Branched-Chain Amino Acids are Associated with Higher Risk of Post-Transplant Diabetes Mellitus in Renal Transplant Recipients

Maryse CJ Osté [1,*], Jose L Flores-Guerrero [1], Eke G Gruppen [2], Lyanne M Kieneker [1], Margery A Connelly [3], James D Otvos [3], Robin PF Dullaart [2], and Stephan JL Bakker [1]

[1] Department of Internal Medicine, Division of Nephrology, University Medical Center Groningen, University of Groningen, 9700 RB Groningen, the Netherlands; j.l.flores.guerrero@umcg.nl (J.L.F.G.); l.m.kieneker@umcg.nl (L.M.K.); s.j.l.bakker@umcg.nl (S.J.L.B.)

[2] Department of Internal Medicine, Division of Endocrinology, University Medical Center Groningen, University of Groningen, 9700 RB Groningen, the Netherlands; e.g.gruppen@umcg.nl (E.G.G.), r.p.f.dullaart@umcg.nl (R.P.F.D.)

[3] Laboratory Corporation of America Holdings (LabCorp), Morrisville, North Carolina, United States of America, connem5@lapcorp.com (M.A.G.), otvosj@labcorp.com (J.D.O.)

* Correspondence: m.c.j.oste@umcg.nl; Tel: +31 50-3713449

Detailed description of the mediation analyses

Mediation analyses can be used to investigate whether the association between a variable of interest and the outcome can be explained by a third variable, which is referred to as a mediator. In mediation analyses the total effect (c) of the variable of interest on the outcome is therefore divided into a direct effect (c′) and an indirect effect through the mediator (a*b), which is the result of the effect of the variable of interest on the mediator (a) and the effect of the mediator on the outcome (b) (Supplementary Figure S3). To establish mediation, we performed mediation analysis in R using the mediation package.[1] First, we estimated the total effect of total BCAA on PTDM (c) using regression analysis. Subsequently we estimated the effect of total BCAA on plasma glucose and HbA1c (a). We estimated the effect of plasma glucose and HbA1c as potential mediators on the PTDM were estimated (b). The indirect effect of plasma glucose and HbA1c was then calculated by computing the product of the 2 regression coefficients of the potential mediator and total BCAA and PTDM (a*b). The magnitude of indirect effects was calculated by dividing the coefficient of the indirect effects by the total effect. All mediation analyses were adjusted for age and sex. Significance of mediation was tested by computing bias-corrected bootstrap CIs with 2,000 repetitions.

1) Tingley D et al. (2014) Mediation: R package for Causal Mediation Analysis. J. Stat. Softw. 59:5. Available via: https://cran.r-project.org/web/packages/mediation/vignettes/mediation.pdf

Supplementary Table S1. Mediating effect of plasma glucose and HbA1c on the association of BCAA with PTDM

	Beta Coefficient (95% CI)*	Proportion Mediated
**Plasma glucose**		
Indirect pathway (ab path)	0.13 (-1.39 – 2.01)	0.3% **
Total effect (ab + c′ path)	-19.09 (-35.65 – -5.37)	
**HbA1c**		
Indirect pathway (ab path)	-58.12 (-205.79 – -6.16)	53% **
Total effect (ab + c′ path)	-103.33 (-347.60 – -8.51)	

Mediation analysis. Beta Coefficient: unstandardized regression coefficient, adjusted for age and sex. Confidence Intervals (95% CI) were bias-corrected and derived after running 2000 bootstrap samples. **The size of the significant mediated effect is calculated as the standardized indirect effect divided by the standardized total effect multiplied by 100. BCAA, branched chain amino acids; PTDM, post-transplant diabetes mellitus.

Supplementary Table S2. Association of BCAAs with all-cause mortality and death-censored graft failure in renal transplant recipients (n=518)

	**Per SD as continuous variable (μmol/L)**		**Lowest tertile vs. higher two tertiles**		
**All-cause mortality**					
**BCAA**					
No. of events	114		42	72	
	HR (95% CI)	*P*	HR (95% CI)	Reference	*P*
Crude	0.91 (0.75-1.11)	0.35	1.22 (0.83-1.78)	1.00	0.31
Model 1	0.89 (0.73-1.08)	0.24	1.29 (0.88-1.91)	1.00	0.19
Model 2	0.90 (0.73-1.12)	0.35	1.22 (0.82-1.82)	1.00	0.33
Model 3	0.89 (0.72-1.10)	0.28	1.25 (0.84-1.86)	1.00	0.28
Model 4	0.90 (0.71-1.12)	0.33	1.32 (0.85-2.04)	1.00	0.22
Model 5	0.91 (0.73-1.15)	0.44	1.24 (0.79-1.92)	1.00	0.35
Model 6	0.89 (0.72-1.10)	0.29	1.22 (0.82-1.83)	1.00	0.33
**Death-censored graft failure**					
**BCAA**					
No. of events	65		25	40	
	HR (95% CI)	*P*	HR (95% CI)	Reference	*P*
Crude	0.86 (0.66-1.12)	0.26	1.32 (0.80-2.18)	1.00	0.27
Model 1	0.86 (0.65-1.13)	0.28	1.32 (0.79-2.21)	1.00	0.30
Model 2	0.92 (0.69-1.23)	0.58	1.34 (0.79-2.27)	1.00	0.28
Model 3	0.83 (0.61-1.13)	0.23	1.35 (0.79-2.31)	1.00	0.28
Model 4	0.86 (0.63-1.18)	0.36	1.14 (0.64-2.04)	1.00	0.65
Model 5	0.92 (0.69-1.23)	0.57	1.03 (0.58-1.84)	1.00	0.92
Model 6	0.87 (0.65-1.17)	0.36	1.13 (0.66-1.92)	1.00	0.65

Cox proportional hazards regression analyses were performed to assess the association of BCAAs with all-cause mortality and death-censored graft failure. Model 1: adjustment for age and sex; model 2: model 1 + adjustment for eGFR, proteinuria, and time since transplantation; model 3: model 2 + adjustment for total cholesterol and triglycerides; model 4: model 2 + adjustment for total energy intake, physical activity, and BMI; model 5: model 2 + adjustment for smoking status and alcohol intake; model 6: model 2 + adjustment for prednisolone dose and trough levels of tacrolimus and cyclosporine. BCAA, branched chain amino acids; PTDM, post-transplant diabetes mellitus; eGFR, estimated glomerular filtration rate

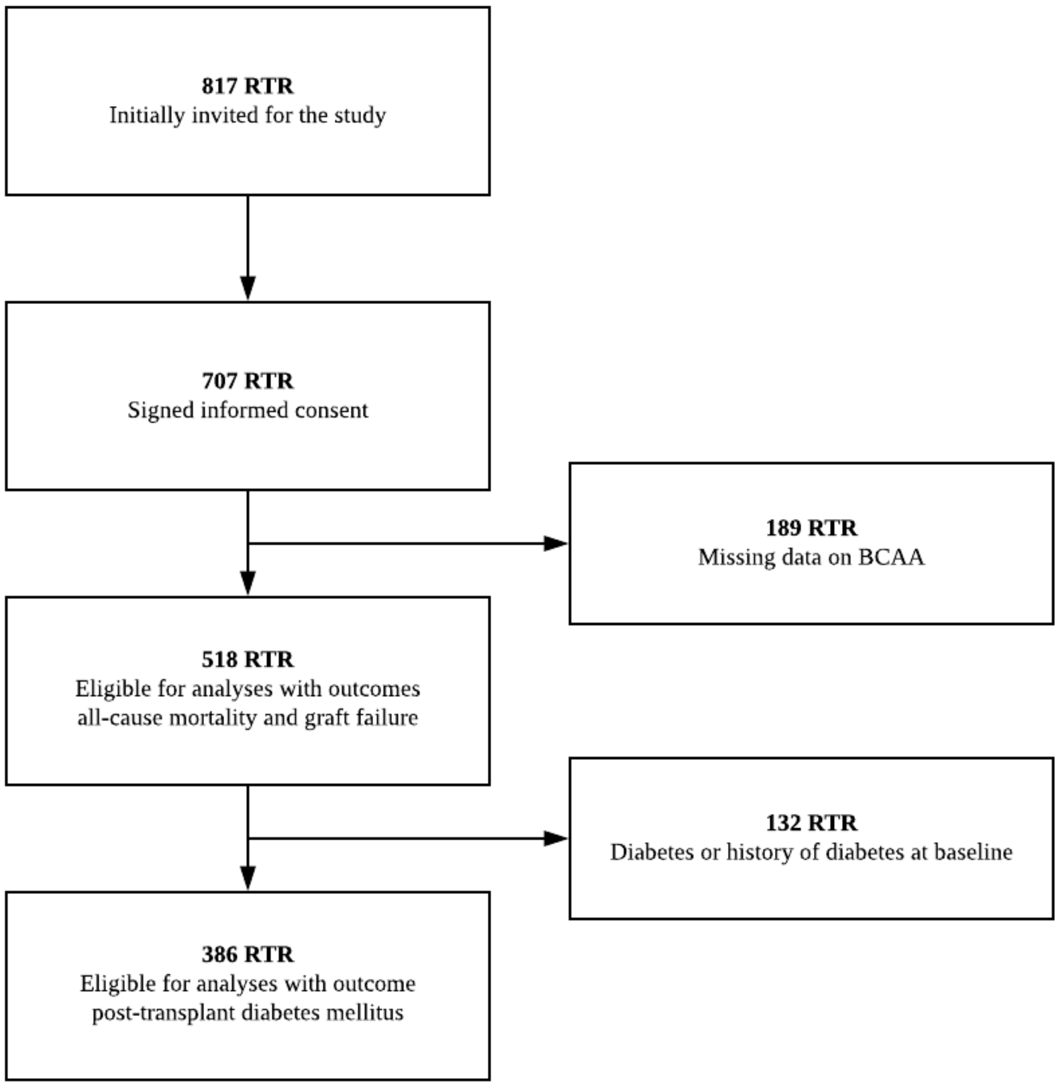

Supplementary Figure S1. Flowchart of the study. RTR, renal transplant recipients.

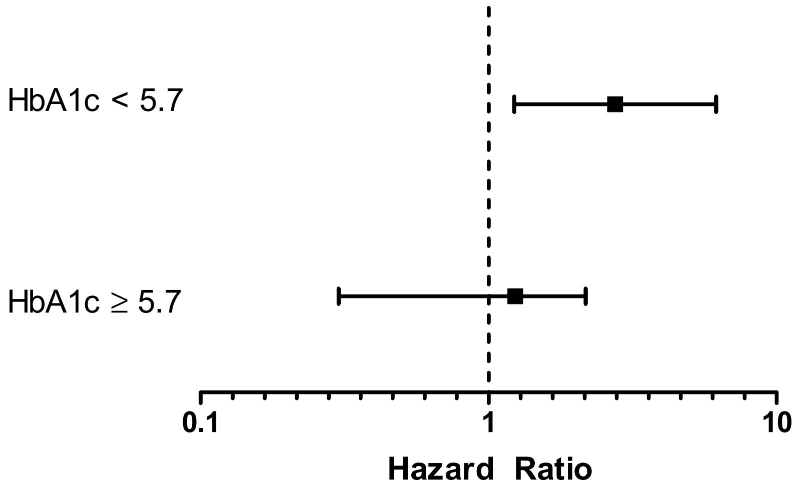

Supplementary Figure S2. Stratified analyses of the association of branched chain amino acids (BCAA) and post-transplant diabetes mellitus (PTDM) in both patients with normal glucose tolerance (HbA1c < 5.7%) and prediabetes (HbA1c ≥ 5.7%) adjusted for age, sex, eGFR, proteinuria, and time since transplantation.

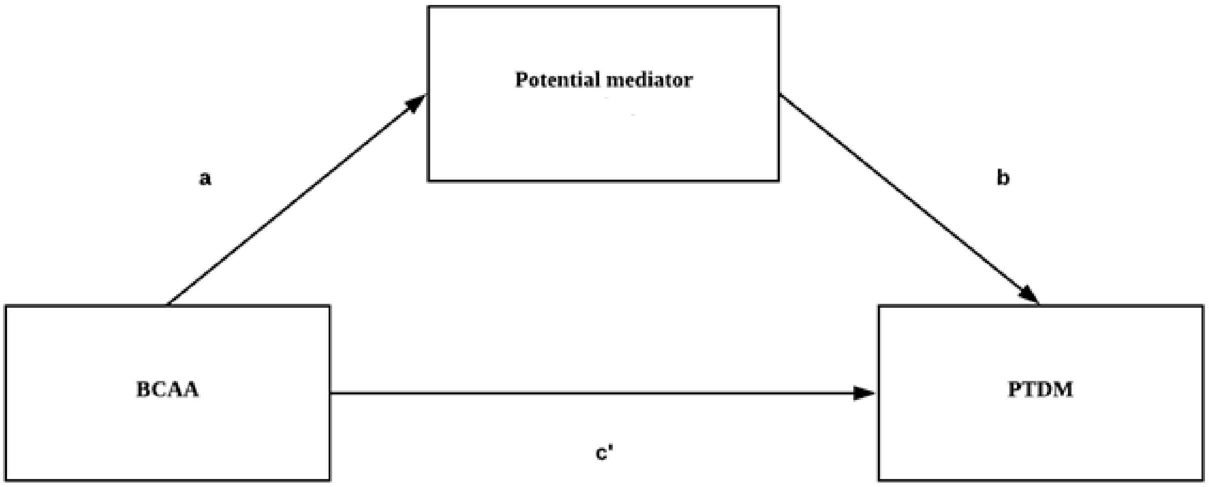

Supplementary Figure S3. Mediation analysis on the association of branched chain amino acids (BCAA) on post-transplant diabetes mellitus (PTDM). a, b and c are the standardized regression coefficients between variables. The indirect effect (through the potential mediator plasma glucose or HbA1c) is calculated as a*b. Total effect (c) is a*b + c′. Magnitude of mediation is calculated as indirect effect divided by total effect.
